# Direct Organogenesis of *Epipremnum aureum* G.S. Bunting for Mass Propagation

**DOI:** 10.3390/plants14213299

**Published:** 2025-10-29

**Authors:** Hai T. Nguyen, Quyet V. Khuat, Thao T. Ninh, Anh T. P. Dang, Le T. Nguyen, Elena A. Kalasnıkova, Abdulmalik A. Batukaev, Rima N. Kirakosyan

**Affiliations:** 1 Department of Plant Biotechnology, Vietnam National University of Agriculture, Gia Lam, Hanoi 12406, Vietnam; ntthao@vnua.edu.vn (T.T.N.); dangphuonganhvandai@gmail.com (A.T.P.D.); nguyenlek57@gmail.com (L.T.N.); 2 Department of Biology and Agricultural Engineering, Hanoi Pedagogical University N°2, Nguyen Van Linh, 32, Phuc Yen 15000, Vietnam; khuatvanquyet@hpu2.edu.com; 3 Centre for Horticultural Science, Queensland Alliance for Agriculture and Food Innovation, The University of Queensland, 306 Carmody Rd., St. Lucia, QLD 4067, Australia; 4 Timiryazev Institute of Plant Physiology, Russian Academy of Sciences, Botanicheskaya, 35, 127276 Moscow, Russia; kalash0407@mail.ru; 5 Chechen Research Institute of Agriculture, Lilovaya St., 1, 366021 Grozny, Russia; batukaevmalik@mail.ru; 6 Department of Fruit and Vegetable Growing and Viticulture, Chechen State University, A. Sheripova, 32, 364061 Grozny, Russia; 7 Department of Biotechnology, Russian State Agrarian University—Moscow Timiryazev Agricultural Academy, Timiryazevskaya, 49, 127550 Moscow, Russia

**Keywords:** micropropagation, organogenesis, 6-benzylaminopurine, kinetin, activated charcoal, acclimatization, cost-effective propagation

## Abstract

Pothos (*Epipremnum aureum* G.S. Bunting), which belongs to the Arum family (Araceae Juss.), can be used for medicinal, ornamental, and pollutant-purifying purposes. Due to the usefulness of pothos, the market demand for this species is increasing. Our study attempts to fill in the shortcomings of previous studies on the effect of activated carbon and plant growth regulators on the ability of shoots to take root in vitro, as well as the effect of inexpensive and readily available materials on the transition of seedlings from in vitro to the greenhouse stage. To evaluate the shooting results, Murashige and Skoog medium (MS) was used, which included 6-benzylaminopurine (BA), kinetin (Kn), α-naphthaleneacetic acid (α-NAA), coconut water, activated carbon, and indole-3-butyric acid (IBA) in various concentrations and combinations. Our results showed that the MS medium with the addition of 2.5 mg/L BA and 1.0 mg/L Kn was optimal for propagation by shoots. In this variant, 2.86 shoots per explant, 1.87 cm of shoot length, and 1.59 leaves per shoot were obtained. Despite the fact that this treatment provided the highest total cytokinin concentration, it was significantly more effective than only BA (2.5 mg/L) and all combinations of BA+α-NAA or Kn+α-NAA. For rooting, the micro shoots obtained on the above medium were transferred to MS + 0.25 mg/L α-NAA + 0.5 g/L AC, which allowed for rooting by 93.33%, 1.93 roots per explant, and root lengths by 2.37 cm. This is higher than with the IBA-based treatment, which led to a shortening of the roots and a reduction in their branching. Acclimatization in a 1:1 mixture (by volume) of loamy garden soil (pH 6.2, 2.1% organic matter) and coconut coir (particle size 0.5–2 mm) gave 75% survival after 40 days. These results have opened up the prospect of developing an effective method for reproducing pothos species in vitro by organogenesis at the lowest cost.

## 1. Introduction

Pothos (*Epipremnum aureum* G.S. Bunting), which belongs to the Arum family (Araceae Juss.), is mostly cultivated in subtropical and tropical climates [1,2]. It possesses beautiful golden-green foliage, with long vines that create a beautiful waterfall effect. In addition to its ornamental value [3], pothos shows important medicinal and pharmacological properties, such as antibacterial, anti-termite, and antioxidant [4,5]. As a solution to air, soil, and water pollution, pothos is well known for the purpose of removing pollutants by metabolizing toxic pollutants or incorporating and sequestering toxic compounds in its tissues [6].

In order to take advantage of these beneficial effects of pothos, in vitro propagation stands out from the crowd for three reasons. Firstly, the extraction and production of natural products from medicinal plants such as pothos by tissue culture will reduce the burden on natural habitats and provide conditions suitable for year-round production [7,8]. Secondly, the difficulty in breeding pothos is due to the challenges of flower induction in nature and even in the greenhouse, resulting in the limitation of diverse cultivars in the market and, hence, not satisfying the demand [9]. Therefore, there is a need to mass produce a huge amount of free-disease plantlets, which are a rich source for plant breeding, and this can be achieved by tissue culture. Thirdly, with the growing interest in using pothos in the office as they can be decorative and purify indoor air as well, in vitro propagation can produce a more compact, uniform size that suits the needs of the customer [10].

While somatic embryogenesis has been extensively used for pothos propagation [9,11,12,13,14,15,16], adventitious shoot organogenesis offers distinct practical advantages for commercial production: faster regeneration, reduced somaclonal variation, and lower dependency on complex hormone regimes [16,17,18,19]. These features are critical for scalable, year-round propagation in limited laboratory spaces—a key requirement for meeting rising market demand. Recent studies have reported adventitious shoot formation in *E. aureum* [11,16,20], yet a complete, reproducible organogenesis-based protocol—from shoot induction through rooting to greenhouse acclimatization—remains lacking. Notably, systematic optimization of cytokinin/auxin combinations for shoot multiplication, as well as cost-effective strategies for rooting and ex vitro transitions, have not been addressed. Therefore, this study was designed to establish an efficient, low-cost micropropagation system for *Epipremnum aureum* via direct organogenesis, specifically optimized for mass propagation and continuous seedling production in minimal spaces, using readily available and inexpensive materials such as coconut water and activated charcoal.

This study was conducted with the aim of developing a cost-effective micro-propagation protocol based on organogenesis for *Epipremnum aureum*, which ensures highly efficient shoot propagation, reliable rooting and successful acclimatization. The developed technology is optimized for year-round mass production in confined laboratory and greenhouse spaces.

## 2. Results

### 2.1. Effect of Different PGRs on Shoot Multiplication of Pothos (Epipremnum aureum G.S. Bunting)

Hormone concentrations were selected based on preliminary dose–response assays and the literature for aroids [12,21,22].

#### 2.1.1. Effect of BA and Kn on Shoot Multiplication of Pothos

**Figure 1:**
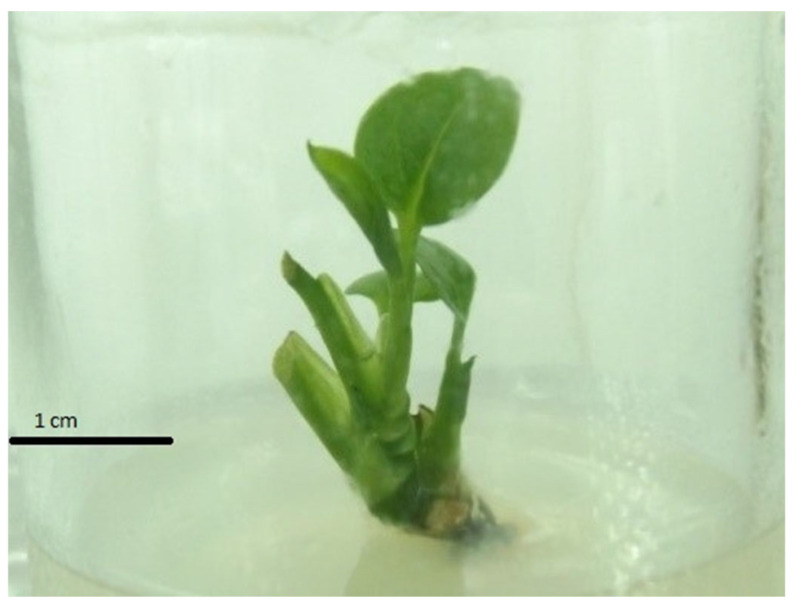
Adventitious shoots of pothos (*Epipremnum aureum* G.S. Bunting).

Adventitious shoots emerged in all treatments after four weeks of culture in a medium supplemented with 2.50 mg/L BA and Kn (from 0 to 1.00 mg/L) (Table 1, Figure 1). The results showed that the combined use of BA and Kn was more effective in creating adventitious shoots than using BA alone. When Kn concentration increased from 0 to 1.00 mg/L, the shoot multiplication rate and the mean length of shoots increased and reached the highest levels of 2.86 shoots per explant and 1.87 cm, respectively. Observations on the mean number of leaves in shoots showed similar trends, except for Kn concentrations at 0.25 mg/L and 0.5 mg/L (Table 1).

**Table 1:** Effect of BA and Kn on shoot multiplication of pothos (*Epipremnum aureum* G.S. Bunting).

Treatment	BA (mg/L)	Kn (mg/L)	Number of Shoots Per Explant	Shoot Length (cm)	Number of Leaves Per Shoot	Shoot Quality
Control	2.50	0	1.86 ± 0.12 c	1.26 ± 0.15 c	1.23 ± 0.14 b	+
2		0.25	1.96 ± 0.05 c	1.42 ± 0.05 bc	1.32 ± 0.07 b	++
3		0.50	2.23 ± 0.20 bc	1.47 ± 0.03 bc	1.14 ± 0.09 b	++
4		0.75	2.53 ± 0.20 ab	1.66 ± 0.10 ab	1.39 ± 0.01 ab	++
5		1.00	2.86 ± 0.11 a	1.87 ± 0.04 a	1.59 ± 0.04 a	+++

Treatment 1—control; Treatments 2–5—experimental variants with increasing concentrations of the specified plant growth regulator(s) as indicated in the columns. The different letters indicate significant differences at *p* ≤ 0.05 according to Duncan’s MRT. Values represent the means ± SE (n = 15 for each treatment). The data have been recorded on a per explant basis and recorded after 4 weeks of culture. (+) Large shoot with bright green leaves; (++) Small shoot with bright green leaves, no or very little yellow spots; (+++) Small shoot with green leaves and yellow spots.

#### 2.1.2. Effect of BA and α–NAA on Shoot Multiplication of Pothos

**Figure 2:**
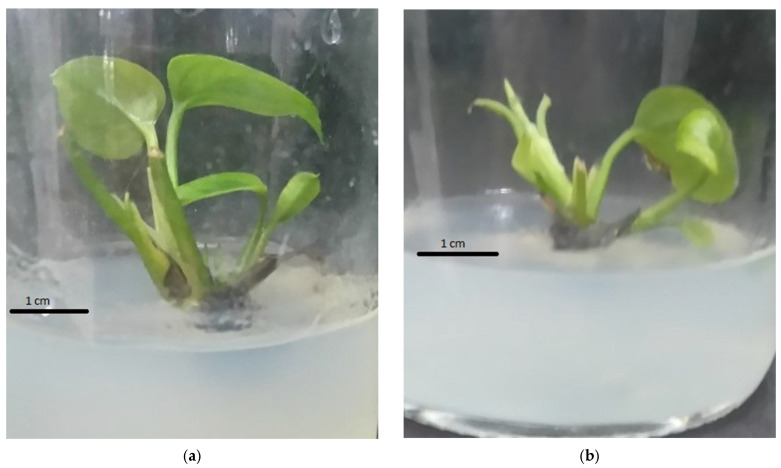

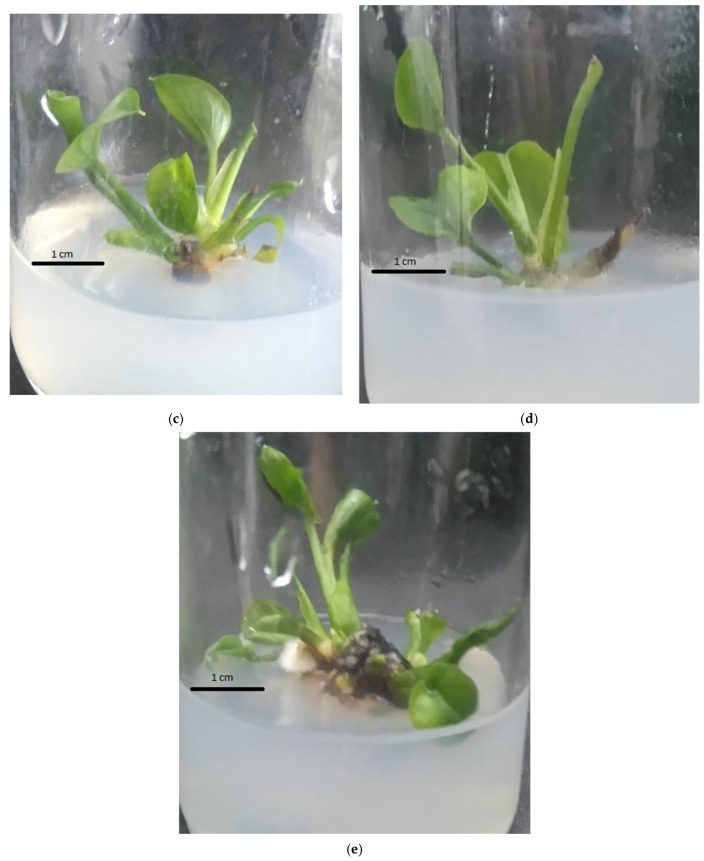
Effect of BA and α-NAA on shoot multiplication of pothos (*Epipremnum aureum* G.S. Bunting). Bunting after 4 weeks of culture: (**a**) MS + 2.50 mg/L BA + 0 mg/L α-NAA; (**b**) MS + 2.50 mg/L BA + 0.25 mg/L α-NAA; (**c**) MS + 2.5 mg/L BA + 0.50 mg/L α-NAA; (**d**) MS + 2.50 mg/L BA + 0.75 mg/L α-NAA; (**e**) MS + 2.50 mg/L BA + 1.00 mg/L α-NAA.

Combining α-NAA with BA proved to be more effective than utilizing BA alone in generating and multiplying adventitious shoots quickly. The mean shoot length and the shoot multiplication rate rose with an increase in α-NAA concentration from 0 to 1.00 mg/L (Table 2). The mean number of leaves per shoot in the treatments did not show significant differences (Table 2). Differences were observed in the morphological traits of adventitious shoots that were obtained from the treatments. In treatment 5, adventitious shoots had large, bright green leaves, but the shoots in the other treatments displayed small, green leaves with yellow spots (Figure 2). Explants in treatments 4 and 5 formed adventitious roots, while the other treatments did not (Figure 2).

**Table 2:** Effect of BA and α–NAA on shoot multiplication of pothos (*Epipremnum aureum* G.S. Bunting).

Treatment	BA (mg/L)	α–NAA (mg/L)	Number of Shoots Per Explant	Shoot Length (cm)	Number of Leaves Per Shoot	Shoot Quality
Control	2.50	0	1.40 ± 0.17 c	1.20 ± 0.08 b	1.23 ± 0.10 a	+
2		0.25	1.53 ± 0.19 bc	1.28 ± 0.03 ab	1.28 ± 0.09 a	++
3		0.50	1.67 ± 0.13 bc	1.37 ± 0.11 ab	1.33 ± 0.06 a	++
4		0.75	2.00 ± 0.08 ab	1.43 ± 0.07 ab	1.39 ± 0.08 a	++
5		1.00	2.47 ± 0.21 a	1.57 ± 0.12 a	1.45 ± 0.06 a	+

Treatment 1—control; Treatments 2–5—experimental variants with increasing concentrations of the specified plant growth regulator(s) as indicated in the columns. The different letters indicate significant differences at *p* ≤ 0.05 according to Duncan’s MRT. Values represent the means ± SE (n = 15 for each treatment). The data have been recorded on a per explant basis and recorded after 4 weeks of culture. (+) Large shoot with bright green leaves; (++) Small shoot with bright green leaves, no or very little yellow spots.

#### 2.1.3. Effect of Kn and α-NAA on Shoot Multiplication of Pothos

Using Kn alone also showed a lower efficiency of adventitious shoot formation than using it in combination with α-NAA (Table 3). In the combined treatment of 2.5 mg/L Kn with 0.75 mg/L α-NAA, the best monitoring indicators were recorded, including the mean number of shoots per explant (2.20 shoots/explant), length of shoot (1.53 cm), and average number of leaves per shoot (1.57 leaves/shoot). In treatments 2 and 3, adventitious roots appeared. The shoots’ morphological traits were largely consistent between treatments, and they all grew and developed well.

**Table 3:** Effect of Kn and α–NAA on shoot multiplication of pothos (*Epipremnum aureum* G.S. Bunting).

Treatment	Kn (mg/L)	α–NAA (mg/L)	Number of Shoots Per Explant	Shoot Length (cm)	Number of Leaves Per Shoot	Shoot Quality
Control	2.50	0	1.27 ± 0.11 c	1.10 ± 0.11 b	1.02 ± 0.15 c	+
2		0.25	1.40 ± 0.08 bc	1.20 ± 0.15 ab	1.33 ± 0.06 ab	++
3		0.50	1.67 ± 0.09 b	1.33 ± 0.08 ab	1.46 ± 0.06 ab	++
4		0.75	2.20 ± 0.17 a	1.53 ± 0.05 a	1.57 ± 0.04 a	++
5		1.00	1.73 ± 0.07 b	1.43 ± 0.04 a	1.22 ± 0.05 bc	++

Treatment 1—control; Treatments 2–5—experimental variants with increasing concentrations of the specified plant growth regulator(s) as indicated in the columns. The different letters indicate significant differences at *p* ≤ 0.05 according to Duncan’s MRT. Values represent the means ± SE (n = 15 for each treatment). The data have been recorded on a per explant basis and recorded after 4 weeks of culture. (+) Large shoot with bright green leaves; (++) Small shoot with bright green leaves, no or very little yellow spots.

#### 2.1.4. Effect of Coconut Water on Shoot Multiplication of Pothos

**Figure 3:**
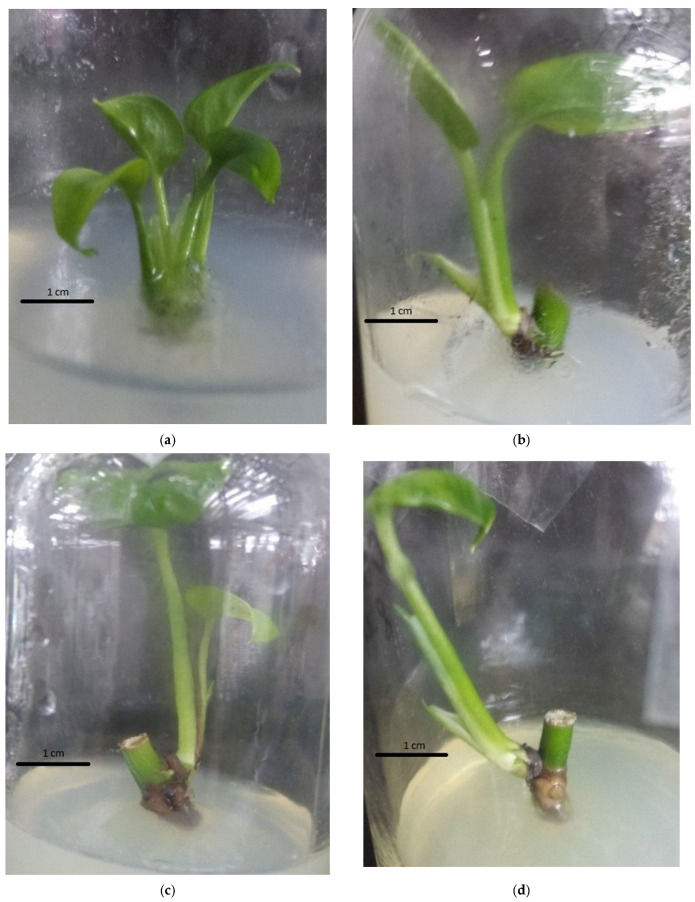

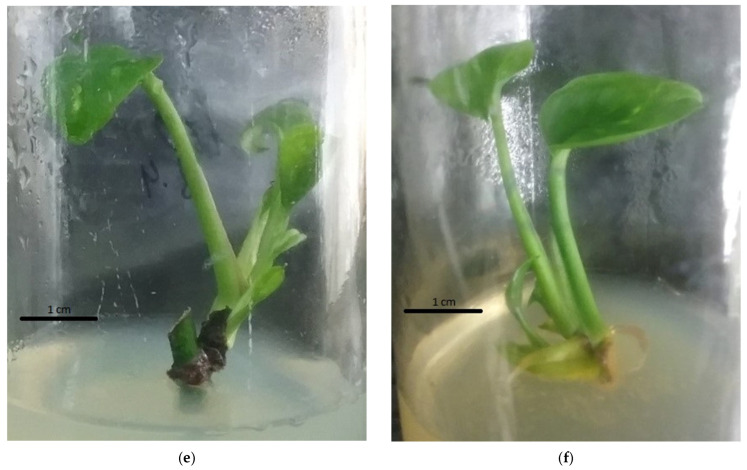
Effect of coconut water on shoot multiplication of pothos (*Epipremnum aureum* G.S. Bunting): (**a**) 0 mL/L coconut water; (**b**) 10 mL/L coconut water; (**c**) 20 mL/L coconut water; (**d**) 50 mL/L coconut water; (**e**) 100 mL/L coconut water; (**f**) 200 mL/L coconut water.

The findings of the study demonstrated that the addition of coconut water to the medium had no discernible effect on adventitious shoot production and rapid multiplication when compared to the control (Table 4). However, shoot length and the mean number of leaves per shoot considerably increased when coconut water was added to the medium. The adventitious shoots in the treatments exhibited similar morphological and development characteristics, such as large, well-developed, and well-growing shoots, as well as bright green leaves (Figure 3).

Among all tested regimens using cytokinins/auxins (Table 1, Table 2 and Table 3), MS + 2.5 mg/L BA + 1.0 mg/L Kn produced the largest number of shoots (2.86 per explant), length (1.87 cm), and number of leaves (1.59), which significantly exceeded the previous variants: only BA (1.86 shoots; Table 1, control), BA+α-NAA (maximum 2.47 shoots at 1.0 mg/L α-NAA; Table 2), Kn+α-NAA (maximum 2.20 seedlings; Table 3).

### 2.2. Effect of Different PGRs on Rooting Efficiency of Pothos (Epipremnum aureum G.S. Bunting)

#### 2.2.1. Effect of α-NAA on Rooting Efficiency of Pothos

The findings of this study demonstrated that adventitious roots formed in all medium treatments (Table 5). The addition of α-NAA to the culture medium showed a significant effect on increasing the adventitious rooting rate of the explant, specifically the rooting percentage and the mean number of roots per explant, both of which increased significantly compared to the control. On the other hand, although the mean length of adventitious roots in treatments supplemented with α-NAA did not show a significant difference compared to the control (except treatment 4), the root quality in these treatments was significantly improved compared to the control.

#### 2.2.2. Effect of IBA on Rooting Efficiency of Pothos

**Figure 4:**
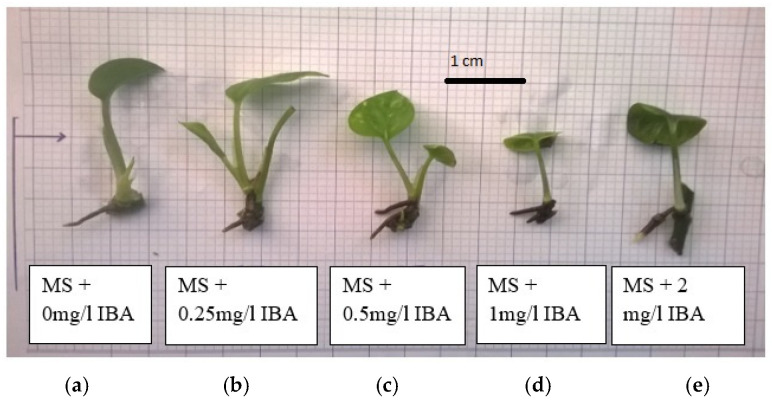
Effect of IBA on rooting efficiency of pothos (*Epipremnum aureum* G.S. Bunting): (**a**) 0 mg/L IBA; (**b**) 0.25 mg/L IBA; (**c**) 0.50 mg/L IBA; (**d**) 1.00 mg/L IBA; (**e**) 2.00 mg/L IBA.

Research results showed that adding IBA to the medium was significantly effective in increasing the rooting percentage and the number of roots per explant (Table 6). The treatment of adding 0.50 mg/L IBA to the medium showed the highest efficiency in creating adventitious roots; specifically, the rooting percentage was 60.00% and the mean number of roots per explant was 1.00. On the other hand, although the mean root length in the IBA-supplemented treatments was significantly lower than the control, the adventitious root quality in these treatments was significantly better than the control, especially in treatment 5 (Figure 4).

#### 2.2.3. Effect of α-NAA and Activated Charcoal on Rooting Efficiency of Pothos

**Figure 5:**
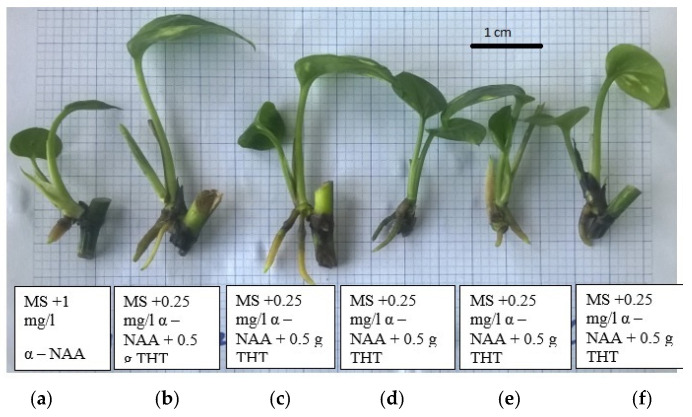
Effect of α-NAA and activated charcoal on rooting efficiency of pothos (*Epipremnum aureum* G.S. Bunting): (**a**) 1.00 mg/L α-NAA; (**b**) 0.5 g/L AC; (**c**) 0.25 mg/L α-NAA + 0.5 g/L AC; (**d**) 0.5 mg/L α-NAA + 0.5 g/L AC; (**e**) 1.00 mg/L α-NAA + 0.5 g/L AC; (**f**) 2.00 mg/L α-NAA + 0.5 g/L AC.

Research results showed that adding activated charcoal to the medium significantly improved rooting percentage and adventitious root length compared to the control using 1.00 mg/L α-NAA (Table 7). However, the medium supplemented with 1.00 mg/L α-NAA showed better effectiveness in increasing the number of roots per explant. In the combined treatment of 0.25 mg/L α-NAA and 0.5 g/L activated charcoal (treatment 3), the best monitoring indicators were recorded; specifically, the rooting percentage was 93.33%, the mean number of roots per explant was 1.93, and the mean root length was 2.37 cm (Table 7). Other combined treatments showed a decrease in adventitious root formation efficiency, but the resulting root quality seemed to be better than in treatment 3 (Figure 5).

With the addition of α-NAA (0.25 mg/L) + AC, the formation of longer, highly branched roots with abundant root hairs is observed (Figure 5c). The use of IBA (0.5 mg/L) causes shortening and thickening of roots with reduced branching (Figure 4c), which is consistent with its well-known role in primary root elongation [23].

### 2.3. Effect of Substrate on the Survival Rate and Development of Pothos (Epipremnum aureum G.S. Bunting)

**Figure 6:**
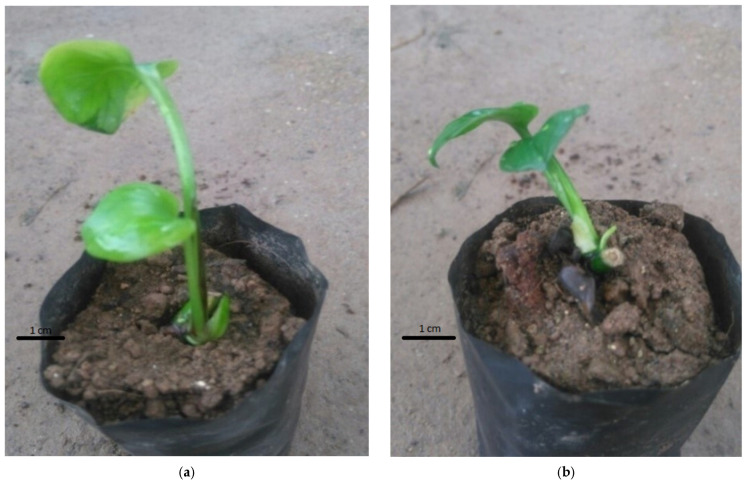

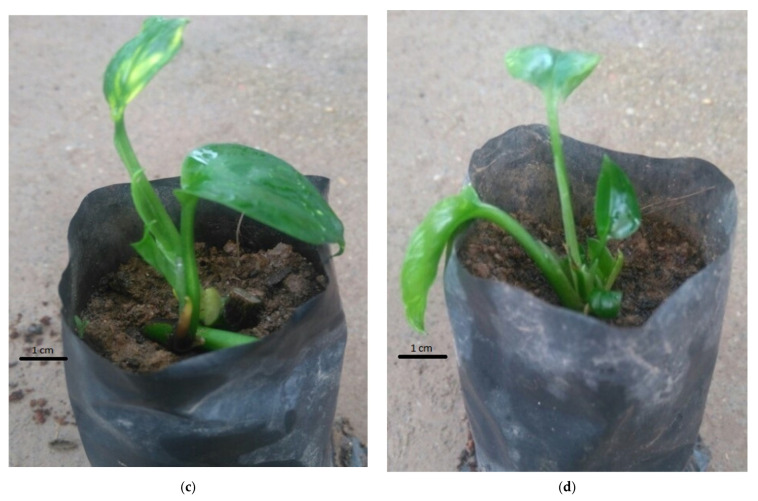
Effect of substrate on the survival rate and development of pothos (*Epipremnum aureum* G.S. Bunting). Bunting after 4 weeks of culture: (**a**) Soil; (**b**) soil–sand (1:1); (**c**) soil–sand (7:3); (**d**) soil–coconut fiber (1:1).

The study’s results showed that the in vitro plantlets with a higher survival rate were planted in soil medium enriched with different proportions of either sand or coconut fiber than the control treatment, which was put in soil alone (Table 8). The best monitoring indicators were recorded in the treatment using soil mixed with coconut fiber in a 1:1 ratio; specifically, the survival rate reached 75.00%, the mean plantlet height was 5.27 cm, and the mean number of leaves per plantlet was 3.39 leaves. All in vitro plantlets were well acclimatized to in vivo conditions, with large, bright green leaves with no or very few yellow spots (Figure 6).

## 3. Discussion

Pothos (*Epipremnum aureum* G.S. Bunting), known as a plant, has many applications in human life, from medicinal and ornamental properties to purifying air pollutants. To extract and produce all year around the beneficial medical compounds and create a massive disease-free and uniform plantlet, tissue culture is a reliable solution. In this study, we conducted an experiment to assess the shooting ability and rooting efficiency in the laboratory and the plant regeneration process in the greenhouse.

In adventitious shoot formation, a combination of auxin and cytokinin show a superior effect on shoot formation [21,24]. Arab et al. [19] concurred with our findings that in order to obtain and rapidly multiply adventitious shoots, shoot length, and number of leaves per shoot, MS medium supplemented with BA and Kn would be preferable to using BA or Kn alone.

In the in vitro propagation of pothos species, previous authors used different combinations to promote shoot formation, shoot length, and the number of leaves per shoot. For example, Qu et al. also used a combination of α-NAA and thidiazuron (TDZ) to obtain 18.2% shoot regeneration frequency and 29.6 shoots/explant after 45 days of culturing [11,12]. The reports of Zhang et al. and Hung showed that MS medium supplemented with a combination of forchlorfenuron (CPPU) and α-NAA was the best for golden pothos shoot regeneration, with shoot induction rates from stem, petiole, and leaf explants of 90, 90, and 68%, respectively [13,15]. In addition, Wang et al. used a combination of BA and α-NAA with the addition of 2,4-dichlorophenoxyacetic acid (2,4-D) to promote callus formation, with the highest being 86% [14]. Zhao et al. used three plant growth regulators, such as CPPU, TDZ, and α-NAA, in calculating the frequency of explants with embryos (%) [9]. This is the first time BA, Kn, and their combination in adventitious shoot formation have been used. In our research, the most suitable medium for the in vitro shoot proliferation stage was MS + 30 g/L sucrose + 8 g/L agar + 2.50 mg/L BA + 1.00 mg/L Kn. The number of shoots per explant was 2.86, the shoot length was 1.87 cm, and the mean number of leaves per shoot was 1.59.

Coconut water is rich in many beneficial compounds for tissue culture, especially in promoting shoot formation and elongation [20]. In our study, MS medium supplemented with 2.50 mg/L BA and 1.00 mg/L Kn combined with 100 mL/L coconut water gave better shoot multiplication coefficients and shoot quality compared to other treatments.

The root is very important, as it can absorb the nutrients as well as keep the plant in place. Before the plant can self-survive, some medium needs to be applied to promote rooting. However, it is considered a challenging step in plant tissue culture [18,22,25].

We developed a complete organogenesis-based protocol for *E. aureum*, building on preliminary reports of adventitious shoot formation [11,20]. Unlike somatic embryogenesis—which requires complex hormone regimes and risks genetic instability [9]—our approach uses simple nodal explants and low-cost additives (coconut water, AC).

The superiority of BA+Kn aligns with findings in Syngonium and Anthurium, where cytokinin combinations enhanced shoot quality [22]. While total cytokinin concentration likely contributed to efficacy, the morphological superiority (larger leaves, reduced vitrification) suggests Kn mitigates BA-induced hyperhydricity. Many previous studies have confirmed the effect of rooting of α-NAA and IBA by regulating many aspects of root development, such as root apical meristem size, root hair elongation, lateral root development, and the formation of adventitious roots [23,26,27,28]. Also, the effect of activated charcoal was seen in the research of Pan et al. and Poniewozik et al. [29,30]. The role of activated charcoal in root growth might be due to its relation to differentially expressed genes in roots and its stimulation of the expression of genes in the phenylpropanoid biosynthesis pathway, in which the protein products promote cell differentiation [30]. It is also possible that activated carbon significantly improves rooting by adsorbing phenolic inhibitors and modulating auxin availability [31]. However, for pothos, Qu et al., Hung, and Zhang et al. did not use any growth regulators for rooting medium in tissue culture [11,13,15].

Our results, the highest rooting percentage, root number per explant, and root length were 93.33%, 1.93 roots, and 2.37 cm, respectively, which were achieved with MS medium combined with 30 g/L saccharose, 8 g/L agar, 0.25 mg/L α-NAA, and 0.5 g activated charcoal. Similarly to the findings of Pan et al., who demonstrated that in the medium supplemented with α-NAA or IAA, the root could form when activated charcoal was present, our research results indicated that α-NAA plus activated charcoal was superior to α-NAA or IBA alone in the rooting percentage [29].

On the other hand, our results showed that high auxin concentrations have negative effects on root parameters. According to Barbez et al., high auxin levels might initiate root apoplastic alkalization, which inhibits cell elongation and enables root bending [32]. The reports of Alarcon et al. and Edelmann also showed similar results [33,34].

Plant establishment in the greenhouse is the last step of tissue culture and is important, especially for the mass production of plants. However, there is not much research on the effect of different kinds of potting media on pothos. For example, Wang et al. and Zhang et al. used peat, vermiculite, perlite, and dolomite to pot “Golden” pothos [13,14]. The mixture containing peat, pine bark, coconut coir, and styrofoam was used for “Jade” pothos in the research of Qu et al. and in Zhao et al. for “Marble Queen” pothos [9,11].

The above mentioned materials for acclimating in vitro plantlets are highly costly and scarce, particularly in Southwest Asia, where pothos are grown most commonly. As a result, items from everyday life like soil, sand, and coconut fiber were used in our study. The difference in the mineral–nutrient ratio and porosity found in the investigated media may account for the better survival rate, plant height, and number of leaves observed in the mixture of soil and coconut fiber as opposed to soil or a mixture of soil and sand. The 75% successful acclimatization in the soil–coconut coir ratio (1:1) emphasizes the value of porous, organic-rich substrates to reduce the shock state during transplantation. The medium of a mixture of sandy soil and coconut fiber soil created more porosity and aeration than the soil medium. Furthermore, the coconut fiber medium has organic natural components that are beneficial for the soil and plants.

## 4. Materials and Methods

Plant material. Mature pothos plants were provided by the Department of Biotechnology, Vietnam University of Agriculture, Vietnam. A stem piece of pothos 1–1.5 cm long with a node, auxiliary bud, and one–two leaves was used as an explant.

Protocol for sterilization of *Epipremnum aureum* included four steps and the fourth step was conducted in a laminar cabinet. In the first step, stem segments were cut into 2–3 cm single nodal segments. In the second step, they were washed under running tap water for 15–20 min and their outer scales were excised by a sharp blade. In the third step, they were soaked in a thin soap solution [1% (*v*/*v*)] for 10 min and washed directly under running tap water. In the fourth step, they were surface sterilized in 70% ethanol for 60 sec, followed by immersion in a 0.1% (*w*/*v*) aqueous mercuric chloride [0.1% (*w*/*v*)] for 15 min. Lastly, after sterilizing with chemicals, they were removed from the damaged tissue at both ends after being rinsed four–five times with sterile distilled water and were placed on solid MS medium [35].

Method. To assess the shooting and rooting ability, plant growth regulators (PGRs) including 6-benzylaminopurine (BA) (Sigma, Schnelldorf, Germany), kinetin (Kn) (Merck, Darmstadt, Germany), α-naphthaleneacetic acid (α-NAA) (Merck, Darmstadt, Germany), coconut water, activated charcoal (AC), and indole-3-butyric acid (IBA) (Merck, Darmstadt, Germany) were added to the Murashige and Skoog (MS) (DUCHEFA, Haarlem, The Netherlands) medium in a variety of concentrations and combinations. The basal medium was MS supplemented with 8 g/L agar and 30 g/L sucrose; pH was adjusted 5.7–5.8 before autoclaving for 20 min at 121 °C, 1.1 atm. The culture condition and experimental design were based on our previous reports, with the temperature maintained at 25 ± 2 °C, photoperiod (16/8 h light/dark), and light intensity of 2300 lux (OSRAM AG, Munich, Germany) [36,37].

Rooting of plantlets: in all rooting experiments, homogeneous micro shoots (1.5–2.0 cm) were used, obtained by adding MS + 2.5 mg/L BA + 1.0 mg/L Kn after four weeks, which ensured stability.

Plantlets’ adaptation to in vivo conditions: in vitro plantlets of pothos were rinsed with running tap water to eliminate the culture medium. After that, these plantlets (n = 15 for each treatment) were treated with 0.5% (*w*/*v*) Bavistin solution (India) (10 min) to prevent fungal contamination and transplanted into plastic pots containing different potting media (sand, soil, and coconut fiber) to access the acclimatization process in the greenhouse. In our study, we used a substrate of the following composition: soil–loamy garden soil (Hanoi, Vietnam), pH 6.2, organic matter 2.1%, total N 0.12%, available P 18 mg/kg, particle size < 2 mm; sand–river sand, pH 6.8, organic matter content is insignificant, granulometry 0.2–1.0 mm; coconut fiber–coconut coir core (Ben Tre, Vietnam, pH 5.8, electrical conductivity 0.4 dS/m, particle size 0.5–2 mm. All substrates were treated in an autoclave (121 °C, 20 min) before use. Data were recorded after 40 days of culture.

Experimental design and statistical analysis: All experiments used a completely randomized design with 15 explants/treatment and three biological replicates (n = 45 total). Mean values of all data were calculated using Microsoft Excel 2013 (Microsoft Corporation, Washington, DC, USA). Analysis of Variance (ANOVA) was performed in Statistica Version 10.0 software (StatSoft, Palo Alto, CA, USA) and means were compared using Duncan’s multiple range test (Duncan’s MRT) at a significance level of *p* ≤ 0.05.

## 5. Conclusions

Due to the usefulness of pothos, the market demand for this species is increasing. This study establishes the first organogenesis-based protocol for *E. aureum*, achieving 93.33% rooting and 75% acclimatization survival using economical, locally sourced materials. The integration of activated charcoal (0.5 g) with low-dose α-NAA (0.25 mg/L) significantly enhances root quality, while soil–coconut fiber substrates enable scalable greenhouse transition. This approach offers a genetically stable, cost-efficient alternative to somatic embryogenesis for commercial pothos production.

## Figures and Tables

**Table 4:** Effect of coconut water on shoot multiplication of pothos (*Epipremnum aureum* G.S. Bunting).

Treatment	Medium	Coconut Water (mg/L)	Number of Shoots Per Explant	Shoot Length (cm)	Number of Leaves Per Shoot	Shoot Quality
Control	2.50 mg/L BA + 1.00 mg/L Kn	0	1.93 ± 0.10 a	1.82 ± 0.16 b	1.17 ± 0.06 b	+
2		10	2.06 ± 0.30 a	3.01 ± 0.07 a	1.24 ± 0.08 ab	++
3		20	2.13 ± 0.09 a	3.24 ± 0.21 a	1.30 ± 0.10 ab	++
4		50	2.26 ± 0.27 a	3.35 ± 0.17 a	1.35 ± 0.04 ab	++
5		100	2.33 ± 0.18 a	3.49 ± 0.14 a	1.40 ± 0.08 a	++

Treatment 1—control; Treatments 2–5—experimental variants with increasing concentrations of the specified plant growth regulator(s) as indicated in the columns. The different letters indicate significant differences at *p* ≤ 0.05 according to Duncan’s MRT. Values represent the means ± SE (n = 15 for each treatment). The data have been recorded on a per explant basis and recorded after 4 weeks of culture. (+) Small and bright green leaves. (++) Large shoots and big, bright green leaves.

**Table 5:** Effect of α–NAA on rooting efficiency of pothos (*Epipremnum aureum* G.S. Bunting).

Treatment	α–NAA (mg/L)	Rooting Percentage (%)	Number of Roots Per Explant	Root Length (cm)	Root Quality
Control	0	40.00 ± 3.46 c	0.40 ± 0.06 c	1.05 ± 0.06 b	+
2	0.25	60.00 ± 2.65 b	0.63 ± 0.05 bc	1.09 ± 0.02 b	++
3	0.50	60.00 ± 2.00 b	0.80 ± 0.08 b	1.14 ± 0.08 b	++
4	1.00	80.00 ± 8.72 a	1.60 ± 0.15 a	1.35 ± 0.03 a	++
5	2.00	66.67 ± 2.96 ab	0.83 ± 0.06 b	1.18 ± 0.05 b	++

Treatment 1—control; Treatments 2–5—experimental variants with increasing concentrations of the specified plant growth regulator(s) as indicated in the columns. The different letters indicate significant differences at *p* ≤ 0.05 according to Duncan’s MRT. Values represent the means ± SE (n = 15 for each treatment). The data have been recorded on a per explant basis and recorded after 4 weeks of culture. (+) Short root; (++) Large root, with root hairs.

**Table 6:** Effect of IBA on rooting efficiency of pothos (*Epipremnum aureum* G.S. Bunting).

Treatment	IBA (mg/L)	Rooting Percentage (%)	Number of Roots Per Explant	Root Length (cm)	Root Quality
Control	0	33.33 ± 3.53 c	0.40 ± 0.08 d	1.28 ± 0.14 a	+
2	0.25	46.67 ± 3.38 b	0.60 ± 0.03 c	0.84 ± 0.06 b	+
3	0.50	60.00 ± 3.06 a	1.00 ± 0.05 a	1.00 ± 0.06 ab	+
4	1.00	53.33 ± 1.20 ab	0.80 ± 0.08 b	0.88 ± 0.07 b	+
5	2.00	53.33 ± 2.19 ab	0.60 ± 0.04c	1.02 ± 0.11 ab	++

Treatment 1—control; Treatments 2–5—experimental variants with increasing concentrations of the specified plant growth regulator(s) as indicated in the columns. The different letters indicate significant differences at *p* ≤ 0.05 according to Duncan’s MRT. Values represent the means ± SE (n = 15 for each treatment). The data have been recorded on a per explant basis and recorded after 4 weeks of culture. (+) Short root. (++) Large root, with root hairs.

**Table 7:** Effect of α-NAA and activated charcoal on rooting efficiency of pothos (*Epipremnum aureum* G.S. Bunting).

Treatment	α-NAA (mg/L)	Activated Charcoal (g/L)	Rooting Percentage (%)	Number of Roots Per Explant	Root Length (cm)	Root Quality
Control 1	0	0	80.00 ± 6.03 b	1.60 ± 0.23 ab	1.19 ± 0.09 c	++
Control 2	0.25	0.5	86.67 ± 2.91 ab	1.13 ± 0.20 b	2.12 ± 0.12 ab	+
3	0.50		93.33 ± 1.20 a	1.93 ± 0.05 a	2.37 ± 0.19 a	+
4	1.00		80.00 ± 1.15 b	1.67 ± 0.23 ab	1.87 ± 0.03 b	++
5	2.00		86.67 ± 2.85 ab	1.53 ± 0.17 ab	1.14 ± 0.06 cd	++
6	0		86.67 ± 2.03 ab	1.33 ± 0.06 b	0.85 ± 0.06 d	++

Treatment 1—control; Treatments 2–5—experimental variants with increasing concentrations of the specified plant growth regulator(s) as indicated in the columns. The different letters indicate significant differences at *p* ≤ 0.05 according to Duncan’s MRT. Values represent the means ± SE (n = 15 for each treatment). The data have been recorded on a per explant basis and recorded after 4 weeks of culture. (+) Short root. (++) Large root, with root hairs.

**Table 8:** Effect of substrate on the survival rate and development of pothos (*Epipremnum aureum* G.S. Bunting).

Treatment	Substrate	Survival Rate (%)	Plantlet Height (cm)	Number of Leaves Per Plantlet	Plantlet Quality
Control	Soil	50.00 ± 3.21 b	4.22 ± 0.19 b	2.00 ± 0.09 b	+
2	Soil + sand (ratio 1:1)	58.33 ± 4.06 b	4.69 ± 0.29 ab	2.33 ± 0.16 b	+
3	Soil + sand (ratio 7:3)	58.33 ± 2.73 b	4.94 ± 0.39 ab	3.00 ± 0.25 a	+
4	Soil + coconut fiber (ratio 1:1)	75.00 ± 2.31 a	5.27 ± 0.14 a	3.39 ± 0.09 a	+

Treatment 1—control; Treatments 2–5—experimental variants with increasing concentrations of the specified plant growth regulator(s) as indicated in the columns. The different letters indicate significant differences at *p* ≤ 0.05 according to Duncan’s MRT. Values represent the means ± SE (n = 15 for each treatment). The data have been recorded after 40 days of culture. (+) All in vitro plantlets were well acclimatized to in vivo conditions, with large, bright green leaves with no or very few yellow spots.

## Data Availability

Data are contained within the article.
